# Antibodies Used to Detect Glaucoma-Associated Myocilin: More or Less Than Meets the Eye?

**DOI:** 10.1167/iovs.19-26843

**Published:** 2019-05

**Authors:** Athéna C. Patterson-Orazem, Raquel L Lieberman

**Affiliations:** School of Chemistry and Biochemistry, Georgia Institute of Technology, Atlanta, Georgia, United States

**Keywords:** antibodies, misfolding, post translational modification

## Abstract

Antibodies are key reagents used in vision research, indeed across biomedical research, but they often do not reveal the whole story about a sample. It is important for researchers to be aware of aspects of antibodies that may affect or limit data interpretation. Federal agencies now require funded grants to demonstrate how they will authenticate reagents used. There is also a push for recombinant antibodies, enabled by phage display technology awarded the 2018 Nobel Prize in Chemistry, which allow for thorough validation and a fixed DNA sequence. Here, we discuss how issues surrounding antibodies are pertinent to detecting myocilin, a protein found in trabecular meshwork and associated with a portion of hereditary glaucoma. Confirmation of myocilin expression in tissues and cell culture has been adopted as validation standard in trabecular meshwork research; thus, a discussion of antibody characteristics and fidelity is critical. Further, based on our basic structural understanding of myocilin architecture and its biophysical aggregation properties, we provide a wish list for the characteristics of next-generation antibody reagents for vision researchers. In the long term, well-characterized antibodies targeting myocilin will enable new insights into its function and involvement in glaucoma pathogenesis.

## Antibodies: Important Research Reagents Invite Closer Scrutiny

Antibodies, highly diverse proteins designed to bind specific targets and assist in combating infections, have been used in research to track specific target antigens across biomedical science since the mid-20th century.^1^ Antibody biologics have also been transformative in their ability to treat a range of diseases.^2^ At the protein level, antibodies consist of constant domains, similar across all antibody subtypes, and variable domains whose diverse complementarity determining regions confer specificity to nearly any desired target antigen (Fig. A). Several subtypes of antibodies are used in research; the most common is immunoglobulin G (IgG). Antibodies used in research are designated polyclonal (pAb), monoclonal (mAb), or recombinant (rAb) based on their basic production method and composition. Traditional pAbs comprise a mixture of antibodies produced by the immune response of an animal to an antigen; the precise mixture varies over time and across individual animals. In contrast, mAbs consist of single antibody “clones” produced by a hybridoma fusion of spleen cells with an immortal myeloma cell line. Still tighter control of the antibody product can be achieved recombinantly by introducing a plasmid containing the DNA sequence encoding the desired antibody into a specialized cell line.

**Figure:**
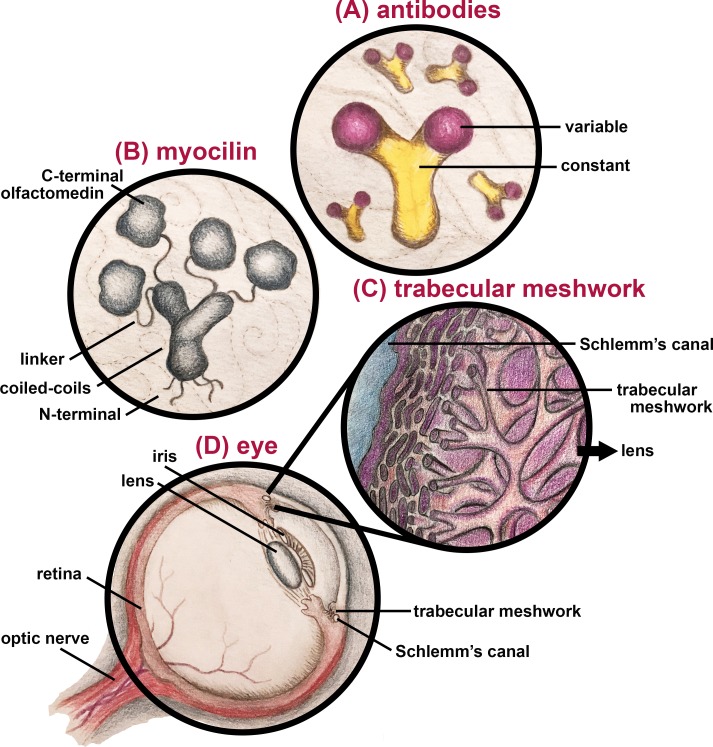
Artistic rendering (not to scale) of antibody, myocilin, TM, and ocular structures. (A) Antibodies display a Y-shape, with constant domains at the base and variable domains at the end of the arms. (B) Myocilin forms a Y-shaped tetramer through its coiled-coil domains, which are attached to four C-terminal olfactomedin structural domains by linkers; the far N-terminal region contains a signal sequence for secretion followed by two cysteine residues that form disulfide bonds. (C) Stylistic representation of the TM tissue where myocilin is expressed at high levels. (D) Basic anatomy of the eye.

Although antibodies are indispensable laboratory research reagents, they are characterized with varying of rigor. Natural variation in titers (e.g., between individual animals or over time) leads to cross-reactivity and batch-to-batch variability of pAbs, and only a low percentage of serum antibodies bind the initial target.^3^ Even antibodies subjected to purification can include a variety of antibody isotypes that target multiple epitopes with a variety of affinities.^4^ The use of hybridomas to express mAbs overcomes some of this variability but, over time, spontaneous mutations introduce a new source of variability.^5^ Antibody fidelity has come under scrutiny after being identified as the likely culprit for irreproducible studies in cancer research, resulting in NIH and other funding bodies requiring more rigorous validation and documentation of research reagents used in the laboratory.^3^,^6^

## Myocilin: Associated With Glaucoma Yet Functionally Elusive

As a case study for how antibodies might not necessarily reveal the whole story of a system, we consider the case of glaucoma-associated myocilin. Wild-type myocilin (Fig. B) is secreted at relatively high levels to the outflow-regulating trabecular meshwork (TM) extracellular matrix (Fig. C) within the eye (Fig. D)^7^; the TM is diseased in most forms of glaucoma. At the protein level, myocilin contains multiple distinct domains: a N-terminal signal sequence for secretion, a structured coiled-coil region for multimerization, a 60 amino acid linker and finally, at its C-terminus, a 250 amino acid β-propeller^8^ olfactomedin (OLF) domain. In its wild-type conformation, the coiled-coil region confers a Y-shaped tetrameric dimer-of-dimers architecture (Fig. B).^9^ If the explicit function of myocilin, or its binding partners, could be readily identified, it would have been already: despite 20 years of research effort in the community, there is no consensus regarding the role of myocilin in normal TM function.

By contrast, our understanding of the role of myocilin in glaucoma is relatively sophisticated. Genetic mutations in myocilin, particularly within its OLF domain, are causative for the ocular hypertension that subsequently leads to early-onset glaucoma.^10^ Myocilin-associated glaucoma is a remarkable example of an autosomal dominant Mendelian inheritance pattern, with affected families harboring unique mutations throughout the world. Overall, nonsynonymous mutations in myocilin are responsible for approximately 3% to 5% of the 70 million open angle glaucoma cases worldwide.^11^ These changes in amino acid sequence result in reduced OLF stability,^12^ protein aggregation and intracellular accumulation in endoplasmic reticulum (ER).^13^,^14^ An anomalous interaction with the ER-resident molecular chaperone Grp94^15^ leads to ER stress^15^–^21^ and cell death,^13^,^17^ hastening the hallmark glaucoma risk factor of increased intraocular pressure.

Variations in myocilin are not a common cause of nonhereditary forms of glaucoma,^22^ as single nucleotide polymorphisms are found in both glaucoma and control populations. However, from a protein perspective, it is easy to envision broader relevance of wild-type myocilin to glaucoma pathogenesis. Most cells deal with proteostasis-related problems, imbalances in protein production, trafficking, and degradation, by undergoing apoptosis. However, long-lived cells such as TM cells^23^ are programmed to avoid cell death^24^ and, consequently, are particularly sensitive to the accumulation of misfolded proteins.^25^ Toxicity of long-lived cells can be triggered by environmental factors as well as destabilizing mutations.^26^ Although wild-type OLF is thermally stable when folded,^12^ it possesses an intrinsic propensity to form a misfolded precipitate of a particular kind called amyloid that is common to many misfolding disorders.^26^ In vitro, purified OLF remains unchanged when incubated at 37°C for weeks at high concentration, but aggregation is readily initiated by adding mimics of glaucoma-associated environmental stressors, including low levels of acid (pH fluctuations), peroxide (oxidative stress), mechanical shear (rocking), and elevated temperature.^27^,^28^ Even though transgenic mice overexpressing wild-type myocilin do not develop glaucoma,^29^ and overexpression alone cannot drive the association of normal myocilin with Grp94,^15^ myocilin driven to misfold by impairing cellular glycosylation interacts with Grp94, culminating in accumulation and toxicity observed for disease-causing variants.^15^ The misfolding susceptibility of wild-type myocilin is further supported by histopathological studies demonstrating its accumulation into punctate bodies in several forms of glaucoma.^30^ Whether accumulated wild-type myocilin contributes to TM damage and outflow resistance remains unknown, but it is clear that our understanding of wild-type myocilin function and dysfunction remains blurry.

## Myocilin Antibodies: We Don't Know What We Cannot Detect

Limitations of the reagents vision scientists have been using to detect myocilin in primary cell culture and tissue samples necessarily affect our contextual understanding of myocilin. For example, early in myocilin research there were reports of an apparent 66-kDa isoform, which was visible by Western blot using multiple myocilin antibodies,^31^,^32^ but was not consistently observed. The jury is still out regarding this 66-kDa species, but it has not been confirmed as myocilin by mass spectrometry,^33^,^34^ and may instead be a closely sequence-related protein^35^ or serum albumin.^33^ Myocilin expression in TM tissue and cell culture has been adopted as a validation standard for the research community,^7^ elevating the need for transparent evaluation of antibody reagents available for the community to use.

Recently, we reported that commercially available myocilin-directed antibodies now used in vision research target epitopes distributed across the protein,^36^ but do not differentiate among folded and misfolded states. Thus, at minimum, we are currently missing information about whether myocilin is properly folded or whether it is adopting a misfolded state in a given sample. Even without destabilizing mutations, we know myocilin is very sensitive to its chemical environment. Beyond OLF being driven to misfold on exposure to environmental stressors discussed above,^27^,^28^ higher-order oligomeric states beyond the native tetramer,^9^ mediated by disulfide bonds at the far N-terminal before the coiled-coils but after its signal sequence, have been detected in several experiments.^37^,^38^ Based on functional studies of olfactomedin family members, all of which contain eponymous OLF domains, posttranslational modifications and protein-protein interactions may be relevant to myocilin detection by antibodies. Cleavage of myocilin has been detected in mammalian cell culture,^39^ in line with a functional feature of other olfactomedin family members.^40^–^42^ This feature has not been established in tissue samples containing myocilin, however, likely because the antibodies used to stain for myocilin only detect one of the cleavage products. Other posttranslational modifications such as phosphorylation or glycosylation may also impede antibody binding, leading to the conclusion that myocilin is not present when it is simply not detectable. Additionally, we expect myocilin, like other olfactomedin family members, to function through complex macromolecular assemblies; for example, interactions between latrophilin OLF and a leucine-rich repeat protein help modulate contacts between nerve cells.^43^ On the other hand, the N-terminal coiled-coil domains of myocilin are “sticky” and predicted to interact with other extracellular matrix proteins.^9^ These attributes of myocilin demand further elucidation, but also experimental consideration including, but not limited to, not relying on any single myocilin epitope to detect myocilin.

## The Future of Myocilin-Directed Antibodies: Our Wish List

To develop the most effective and reliable antibody reagents, consideration of the molecular properties of both antigens and antibodies is key. Understanding how an antibody interacts with its target antigen allows researchers to engineer antibodies for their own purposes, using a selection of epitopes enlightened by structural understanding of the antigen. In addition, high-throughput sequencing and efficient in vitro methods such as phage display are now available to identify, design, and optimize identified antibodies. Indeed, the 2018 Nobel Prize in Chemistry shared by Arnold, Smith, and Winter recognizes contributions to the directed evolution and phage display strategies, which represent landmark developments in protein engineering methodology directly applicable to antibodies.

Knowledge of myocilin structure and misfolding should facilitate the development of new antibody tools to study the protein in unprecedented detail. Ideal antibodies would harbor the following characteristics: (1) detect a variety of unique epitopes throughout the myocilin protein, (2) be conformationally specific to a well-defined state of myocilin (folded, misfolded), (3) be recombinant to enable high-quality control standards maintained by DNA sequencing^6^ and not constitute a limited reagent, and (4) harbor cross-reactivity for myocilin from multiple species, to streamline antibody use across the vision research community.

Beyond their application in validating primary human TM cells and tissues,^7^ a new suite of antibodies targeting deliberate molecular aspects of myocilin would reveal currently inaccessible specifics of myocilin in any given research sample of interest. Such information should lead to a better understanding of myocilin in normal physiology, as well as the role of myocilin misfolding in normal eye aging, glaucoma, and possibly other scenarios in the body. In turn, the community will develop a more nuanced molecular picture of the TM, a complex and fascinating eye tissue that is regularly subjected to numerous chemical and biomechanical insults, unveiling new targets for antiglaucoma therapies.
